# Reactive Oxygen Species, SUMOylation, and Endothelial Inflammation

**DOI:** 10.1155/2012/678190

**Published:** 2012-09-06

**Authors:** Nhat-Tu Le, James P. Corsetti, Janet L. Dehoff-Sparks, Charles E. Sparks, Keigi Fujiwara, Jun-ichi Abe

**Affiliations:** ^1^School of Medicine & Dentistry, Aab Cardiovascular Research Institute, University of Rochester Medical Center, 601 Elmwood Avenue, Box CVRI, Rochester, NY 14642, USA; ^2^School of Medicine and Dentistry, the Department of Pathology and Laboratory Medicine, University of Rochester Medical Center, 601 Elmwood Avenue, Box 608, Rochester, NY 14642, USA

## Abstract

Although the exact mechanism through which NADPH oxidases (Nox's) generate reactive oxygen species (ROS) is still not completely understood, it is widely considered that ROS accumulation is the cause of oxidative stress in endothelial cells. Increasing pieces of evidence strongly indicate the role for ROS in endothelial inflammation and dysfunction and subsequent development of atherosclerotic plaques, which are causes of various pathological cardiac events. An overview for a causative relationship between ROS and endothelial inflammation will be provided in this review. Particularly, a crucial role for specific protein SUMOylation in endothelial inflammation will be presented. Given that SUMOylation of specific proteins leads to increased endothelial inflammation, targeting specific SUMOylated proteins may be an elegant, effective strategy to control inflammation. In addition, the involvement of ROS production in increasing the risk of recurrent coronary events in a sub-group of non-diabetic, post-infarction patients with elevated levels of HDL-cholesterol will be presented with the emphasis that elevated HDL-cholesterol under certain inflammatory conditions can lead to increased incidence of cardiovascular events.

## 1. Introduction

Small ubiquitin-related modifier (SUMO) proteins are ubiquitously expressed in eukaryotic cells [1–4] and are highly conserved from yeast to human. They are attached to specific lysine residues on their substrates through the SUMOylation process, which is catalyzed by E3-like ligase enzymes (E3 SUMO) ligase enzymes. Interestingly, recent studies have revealed that the protein inhibitor of activated STATs (PIAS) proteins, which are initially identified as negative regulators of cytokine signaling that inhibit the activity of STAT transcription factors, act as E3 SUMO ligase enzymes. Because the SUMO E3 ligase activity and the transcriptional coregulator activity are functionally correlated in most cases, the PIAS/SUMO complex appears to be critical for regulating transcriptional activity. Our group has reported the crucial role of reactive oxygen species (ROS) in SUMOylation and possible effects of protein SUMOylation on endothelial function. In this paper, we will discuss some key findings that have elucidated the role for the PIAS/SUMO complex in the transcriptional regulation. Although SUMOylation is implicated in a variety of cellular processes, this paper will focus on the effect of ROS-mediated SUMOylation on endothelial inflammation. In addition, we will also discuss the clinical evidence for the critical involvement of ROS production on the progress of cardiovascular disease (CVD), especially in the patient population with high levels of HDL cholesterol and C-reactive protein (CRP).

## 2. SUMOylation

Among posttranslational modifications, ubiquitination and SUMOylation are unique because they require the covalent interaction between ubiquitin (ubiquitination) and SUMO (SUMOylation) to their protein substrates instead of the addition of a functional group such as a phosphate, acetate, lipid, or carbohydrate. Ubiquitination and SUMOylation are analogous. Although the structures of ubiquitin (a 76-amino acid polypeptide) and SUMO (a 101-amino acid polypeptide) are similar, they share only ~18% sequence homology [5, 6]. 

**Figure 1:**
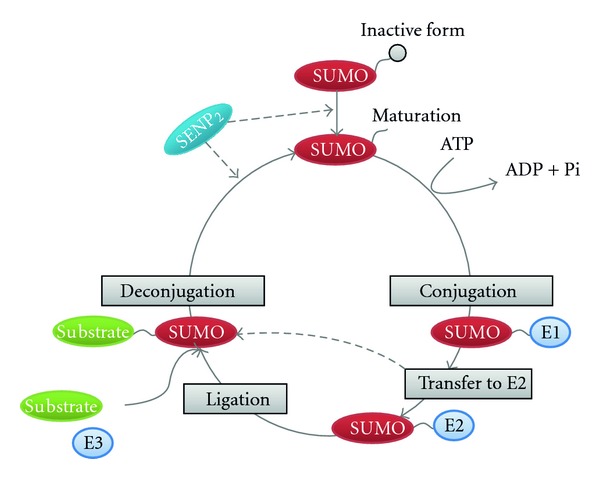
SUMOylation process. Protein SUMOylation consists of deconjugation and conjugation pathways. SUMO-conjugation requires three classes of enzymes (E1→ E2 → E3). SUMO-deconjugation requires the sentrin/SUMO-specific proteases (SENP2).

 SUMOylation is a dynamic and reversible process regulated by both conjugation and de-conjugation enzymes via a three-step process. First, free SUMO is covalently linked to the E1 activating enzyme in an ATP-dependent reaction. Next, SUMO is transferred from the E1 enzyme to the E2 conjugating enzyme. Finally, interaction between the E2 and the E3 ligase enzymes allows the E3 ligase enzyme to initiate the transfer of SUMO from the E2 enzyme to a lysine residue on the substrate [7, 8]. The regulatory mechanism of SUMOylation is analogous to that of ubiquitination, but the two processes employ different sets of enzymes (Figure 1) [6]. SUMOylation is a part of important regulatory mechanisms that modify proteins in the nucleus and regulate multiple cellular processes such as nucleo-cytoplasmic signal transduction [9], stress responses, subcellular localization of proteins, protein-protein interactions, protein-DNA interactions, and transcriptional activity of transcription factors [10].

## 3. SUMO E3 Ligase-PIAS Family of Proteins

Attempts to isolate proteins that regulate the signal transducer and activator of transcriptions (STATs) have identified protein inhibitors of the activated STAT (PIAS) family [11, 12]. The PIAS protein family consists of four members: PIAS1, PIAS2 (PIASx), PIAS3, and PIAS4 (PIASy) [13]. STATs and NF-*κ*B, the two important transcription factor families that are activated in response to a wide range of inflammatory stimuli to regulate multiple cellular processes [10, 14, 15], are negatively regulated by both PIAS1 and PIAS4 [16]. In addition, PIAS proteins also display SUMO E3 ligase activity and promote SUMOylation [13]. 

### 3.1. Structure of PIAS Proteins

**Figure 2:**
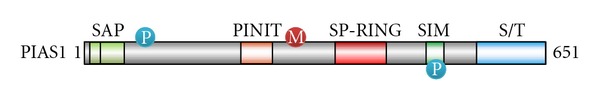
Schematic structure of PIAS1.

The size of mammalian PIAS proteins varies from 510 (PIAS4) to 651 (PIAS1) amino acids. They share highly homologous sequences. Overall, five different motifs on PIAS proteins have been characterized: (1) an N-terminus SAP motif (scaffold attachment factor-A/B, acinus and PIAS), (2) a PINIT motif, (3) a RING-type zinc-binding motif (SP-RING), (4) an SIM motif (SUMO-interacting motif), and (5) a serine/threonine-rich C-terminus region (S/T). The N-terminus SAP and the middle SP-RING motif are the most conserved regions. The C-terminus S/T motif is the least conserved region (Figure 2) [13, 17]. The SP-RING motif, although lacking two zinc-coordinating cysteines [18] compared to the classical RING domain, is suggested to resemble the classical RING domain, which has ligase function. The PINIT motif, which regulates PIAS nuclear retention, also plays a role in PIAS SUMO E3 ligase function for some substrates [19, 20]. On the contrary, the SIM motif is not required for PIAS SUMO E3 ligase activity, despite its ability to interact noncovalently with SUMO proteins [13]. 

### 3.2. PIAS as a Transcriptional Repressor of NF-*κ*B and STAT1

NF-*κ*B is an important transcription factor that regulates many inflammatory genes such as cytokines, chemokines, and adhesion molecules that play major roles in atherosclerosis. Tumor necrosis factor-*α* (TNF-*α*) is a key inflammatory cytokine involved in the progression of atherosclerosis by activating NF-*κ*B signaling [21]. Through the canonical NF-*κ*B pathway, TNF-*α* activates IKK to phosphorylate and degrade I*κ*B, releasing NF-*κ*B into the nucleus where it can activate the transcription of inflammatory genes. Biochemical and genetic studies have demonstrated that PIAS1 negatively regulates this pathway by interacting with NF-*κ*B-p65 to repress its transcriptional activity, thus downregulating the expression of TNF*α*-induced genes [22]. 

PIAS1 has also been shown to bind directly to STAT1 and repress STAT1 transcriptional activity. PIAS1^−/−^ mice are more sensitive to inflammatory responses mediated by interferon-*γ* (IFN-*γ*) or interferon-*β* (IFN-*β*) [12, 22, 23], and are hypersensitive to lipopolysaccharides (LPS) that induces endotoxic shock [22]. The STAT1 and NF-*κ*B activities are increased in PIAS1^−/−^ mice. In response to inflammatory stimuli, PIAS1 is rapidly phosphorylated at Ser-90, which is required for PIAS1-mediated inhibition of STAT1 or NF-*κ*B. The phosphorylation of PIAS1 at Ser-90 is mediated by IKK*α* during TNF*α*-induced inflammation and blocks its NF-*κ*B repressor function, which acts as a negative feedback mechanism on the TNF*α*-IKK*α*-NF-*κ*B signaling pathway [24]. 

### 3.3. PIAS as a SUMO E3 Ligase

In NF-*κ*B activation, the regulatory subunit NF-*κ*B essential modulator (NEMO/IKK*γ*) of the cytoplasmic I*κ*B kinase complex (IKK complex) plays a central role [14]. SUMO-1 modification of NEMO/IKK*γ* is required for NF-*κ*B activation in response to genotoxic stress inducers [25]. Attempts to identify a SUMO E3 ligase that is critical for the SUMO-1 modification of NEMO have indicated the involvement of PIAS4 in this process. PIAS4 interacts with NEMO and preferentially stimulates site-selective modification of NEMO by SUMO-1. Subsequently, the activation of NF-*κ*B is enhanced [26].

The activation of NF-*κ*B can be antagonized by peroxisome proliferator-activated receptor *γ* (PPAR*γ*). PPAR*γ* is a target for SUMO-1 modification. PPAR*γ* agonists induce ligand-dependent conjugation of SUMO-1 to PPAR*γ*. SUMOylation of PPAR*γ* mainly occurs at the Lys-107 residue, resulting in significant inhibition of PPAR*γ* transcriptional activity [27]. When adenoviral vector expressing PPAR*γ*-K107R was delivered into the rat carotid arteries after balloon injury, a significant decrease in neointimal formation was noted, compared to arteries treated by wild type or control vector [28]. Thus, PPAR*γ* SUMOylation at Lys-107 residue not only downregulates its transcriptional activity but also increases neointima formation. SUMOylation of PPAR*γ* is mediated by PIAS1 SUMO E3 ligase. Because PIAS1 can participate directly in the inhibition of LPS-induced NF-*κ*B-mediated inflammatory gene activation, PIAS1 has two different pathways to inhibit NF-kB activation [10, 27]. 

## 4. ROS-Mediated ERK5 SUMOylation ****and Inflammation

### 4.1. ERK5 as a key Factor to Inhibit Endothelial Inflammation

**Figure 3:**
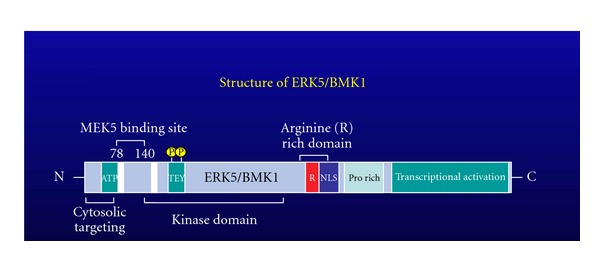
Schematic structure of ERK5.

The mitogen-activated protein kinase (MAPK) is protein kinase that is activated by the redox and hyperosmotic stresses, growth factors, and pathways involving certain G-protein-coupled receptors [29]. Extracellular-signal-regulated kinase 5 (ERK5), or BMK1 (big MAPK1), is the newest member of the MAPK family. The human *erk5* gene (or *MAPK7*) is located on chromosome 17p11.2, extends 5.79 kb, and encodes a protein of 816 amino acids with a predicted molecular mass of 98 kDa (Figure 3) [30]. The ERK5 kinase domain (a.a. 78–406) is on its NH_2_-terminus. On the ERK5 NH_2_-terminus, amino acids 1–77 are important for cytoplasmic targeting, amino acids 78–139 are required for interaction with MEK5, and amino acids 140–406 are important for oligomerization [31, 32]. ERK5 shares approximately 66% sequence homology with ERK1/2 within the kinase domain, which contains the TEY dual phosphorylation motif on its activation loop. However, the long ERK5 COOH-terminus (~400 a.a.) makes ERK5 unique among the MAPK family members. The ERK5 COOH-terminus contains a nuclear localization signal (NLS) (a.a. 505–539) and two proline-rich domains (a.a. 434–465 and 578–701) that are suggested to serve as binding sites for SH3 (Src homology 3)-domain-containing proteins [32, 33]. In addition, the ERK5 COOH-terminus also contains a myocyte enhancer factor 2 (MEF2)-interacting region (a.a. 440–501) and two transcriptional activator domains (a.a. 664–789) that regulate MEF2 transcription factor activity [31]. Therefore, ERK5 has not only kinase but also transcriptional activity. The ERK5 NH2-terminus works as a negative regulator of these transcriptional activator domains. The upstream kinase that phosphorylates ERK5 has been identified as MEK5*α* [33, 34]. When activated, ERK5 releases its NH_2_-terminus inhibitory effect, enabling transcriptional activity of the COOH-terminus. Therefore, ERK5 transcriptional activity is regulated by an intramolecular interaction [35]. However, the ERK5 COOH-terminus tail (a.a. 684–806) also possesses a basal transcriptional activity even without the activation induced by MEK5*α* kinase. Similar to other MAPK family members, ERK5 plays a significant role in cell growth and differentiation. Nevertheless, emerging evidence suggests ERK5's unique functional characteristics. 

It has been well studied that steady laminar flow (s-flow) generates a frictional dragging force on the endothelium surface (called fluid shear stress), which is known to possess anti-inflammatory and antiatherosclerotic effects and to protect endothelial cells (ECs) from becoming dysfunctional [36, 37]. ERK5 is strongly activated by s-flow through the activation of its upstream MEK5*α*. Once activated, the arginine-rich middle region of ERK5 binds the hinge-helix region of PPAR*γ*1, thus increasing PPAR*γ*1 transcriptional activity [35]. Moreover, the activation of MEK5*α*/ERK5 increases transcriptional activity of MEF2, a crucial component of the transcriptional machinery required for regulating Krüppel-like factor-2 (KLF2) expression. 

KLF2 is a mechanoactivated transcription factor that induces vasoprotective, anti-thrombotic, and anti-inflammatory responses to s-flow [38–40]. MEF2 binds the endogenous KLF2 promoter [41] and increases its activity. The increased KLF2 activity results in an orchestrated regulation of endothelial transcriptional programs that control inflammation, thrombosis/hemostasis, vascular tone, and blood vessel development [41]. Because KLF2 enhances the expression of endothelial nitric oxide synthase (eNOS) and reduces the expression of cytokine-mediated adhesion molecules [39, 41, 42], s-flow-mediated ERK5/MEF2/KLF2 induction leads to the upregulation of eNOS and the downregulation of endothelial inflammation [39, 41, 42]. Thus, s-flow-mediated ERK5 activation plays a critical role in regulating PPAR*γ* as well as KLF2, which subsequently inhibits endothelial inflammation and maintains normal vascular reactivity.

**Figure 4:**
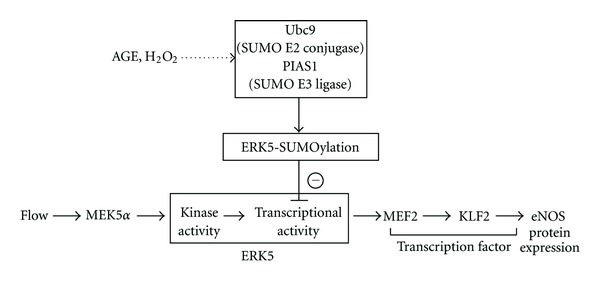
A signaling scheme describing the relationship between the laminar flow-mediated ERK5/MEF2/KLF2/eNOS pathway and H_2_O_2_ or AGE-mediated ERK5-SUMOylation (License number: 2860201127159, date: Mar 01, 2012).

The blood flow pattern inside blood vessels is complex, and different flow patterns activate different signaling events. While s-flow is vessel protective, there is a strong correlation between localized atherosclerotic plaque development and regions of the endothelium exposed to disturbed flow (d-flow) that are found at vessel curvatures, bifurcations, and branches. It has been shown that d-flow increases endothelial apoptosis and inflammation by promoting ROS production, which reacts with NO to form peroxynitrite and induces proatherogenic responses in ECs [43, 44]. Previously, we have reported that ROS induces endogenous ERK5 SUMOylation at Lys-6 and Lys-22 and that this SUMOylation inhibits s-flow-mediated ERK5 transcriptional activity in ECs. We have also found that d-flow can increase ERK5 SUMOylation (unpublished data). The inhibition of ERK5 transcriptional activity by ERK5 SUMOylation results in an inhibition of s-flow-mediated KLF2 promoter activity, subsequently inhibiting the KLF2 and eNOS protein expression in ECs (Figure 4) [45]. Interestingly, the negative regulation of ERK5 transcriptional activity by SUMOylation is independent of ERK5 phosphorylation as well as kinase activation [45]. Inhibition of ERK5 SUMOylation by constitutively active (CA)-MEK5*α* is independent of ERK5 kinase activity, but is dependent on the binding between MEK5 and ERK5 [46]. Thus, our observations imply a crucial role of ERK5 SUMOylation in ROS-mediated ERK5 transcriptional repression, which may contribute to EC inflammation and dysfunction [45]. 

## 5. p53-SUMOylation and Inflammation

### 5.1. D-Flow Induces ROS Production and Increases Endothelial Cell Apoptosis via PKC*ζ*-PIAS4-p53 SUMOylation

ROS functions as a second messenger for various biological responses. NADPH oxidase (Nox) has been identified as the major ROS producing enzyme in blood vessels in response to d-flow [47]. Our recent study has shown that the activation of protein kinase C*ζ* (PKC*ζ*) by d-flow-mediated ROS induces EC apoptosis by regulating p53 [43]. PKC*ζ* activation has been reported in the lesser curvature of the aortic arch in porcine aorta [48], suggesting a proatherogenic role of PKC*ζ* and a possible correlation between d-flow and the activation of this kinase. To verify the potential effect of shear stress on PKC*ζ* activation in ECs *in vitro*, we exposed ECs to different flow patterns and found increased PKC*ζ* activation by d-flow, but not by s-flow [43]. Indeed, activation of PKC*ζ* mediated by d-flow plays a critical role in endothelial apoptosis *in vitro* [43, 49, 50]. 

The p53 tumor repressor is activated by various cellular stresses. It is a key regulator of cell death. p53 plays a proapoptotic role in both transcription independent and dependent manners. On one hand, p53 directly interacts with the B cell lymphoma/leukemia-2 (Bcl-2) protein family members, Bcl-xL and Bcl-2, thus antagonizing their antiapoptotic function by stabilizing the outer mitochondrial membrane [50]. This is a transcription independent mechanism. On the other hand, p53 promotes the transcription of several proapoptotic genes such as p53 upregulated modulator of apoptosis (PUMA) and Bad [50–55]. In most cases, p53 antiapoptotic effect is attributed to its nuclear localization, because nuclear p53 can protect cells from apoptosis, especially under low stress conditions [56, 57]. In our study, we observed the antiapoptotic nuclear localization of p53 in ECs in areas exposed to s-flow. In contrast, d-flow increases p53 nuclear export, which in turn increases p53-Bcl-2 interaction, and subsequently antagonizes antiapoptotic effect of Bcl-2, resulting in enhanced EC apoptosis [43]. 

p53 nuclear export is positively regulated by its SUMOylation, which involves PIAS4 as a SUMO E3 ligase enzyme [52]. Interestingly, d-flow-mediated PKC*ζ* activation also regulates p53 nuclear export via SUMOylation. Once activated, the PKC*ζ* C-terminus kinase domain (a.a. 401–587) interacts with PIAS4 at the SP-RING domain. This binding between PKC*ζ* and PIAS4 is required for p53 SUMOylation, which then increases p53 nuclear export, enhances p53-Bcl2 interaction, and consequently EC apoptosis [43]. Although the role of vascular p53 in either promoting or dampening the process of atherosclerosis remains controversial, we suggest that the PKC*ζ*-PIAS4-p53 SUMOylation pathway should be investigated in the context of the pathogenesis of atherosclerosis. 

### 5.2. The Role of p53 in KLF2 Regulation

In several cell types, p53 stimulates inflammatory signaling and inflammatory gene expression [58, 59]. Recently, it has been reported that endothelial p53 promotes EC dysfunction and impairs EC-dependent NO production by suppressing the expression of KLF2. By binding to a 27-bp sequence on the KLF2 promoter, p53 increases the hypoacetylation of histone H3 on KLF2 promoter and thus decreases KLF2 expression [60]. Since p53 SUMOylation can increase p53 expression by decreasing its degradation, it is also possible that p53 SUMOylation can increase EC inflammation via regulating KLF2 expression. However, further studies are necessary to elucidate this hypothesis.

## 6. MK2-SUMOylation and Inflammation

The MAPK-activated protein kinase 2 (MK2) is a direct substrate of p38 MAPK-*α* and -*β*. p38 MAPK binds to a docking site on the C-terminus of MK2 and subsequently phosphorylates MK2 at different regulatory sites [61–63]. The phosphorylation of MK2 mediated by p38 MAPK results in MK2 nuclear export and serves a dual function. First, it leads to an increase in MK2 kinase activity, which in turn results in the phosphorylation of its substrates such as heat shock protein 25 (HSP25), heat shock protein 27 (HSP27), tyrosine hydroxylase, Cdc25B/C, and leukocyte-specific protein 1 [64–68]. Second, it determines the nuclear export of p38 MAPK [69]. In addition to determining the subcellular localization of p38 MAPK, MK2 has a role in stabilizing it. Notably, MK2 kinase activity is not required for p38 MAPK stabilization [70]. 

TNF-*α* has also been shown to activate the MK2-HSP27 pathway to induce actin filament remodeling [71, 72]. As a mechanism by which TNF-*α* mediates actin filament remodeling via MK2-HSP27, we have suggested MK2 SUMOylation, which is a novel mechanism for regulating actin filament dynamics in ECs. The TNF-*α* mediated-MK2 SUMOylation occurs mainly at lysine (K)-339. The MK2-K339R SUMOylation defective mutant exhibits an increased kinase activity and a sustained phosphorylation level of HSP27 compare to WT-MK2, suggesting the inhibitory effect of MK2 SUMOylation on its kinase activity and subsequent phosphorylation of HSP27. The alignment of ECs in response to laminar flow due to the increase in HSP27 phosphorylation and the subsequent increase in actin filament remodeling is significantly increased in the MK2-K339R SUMOylation defective mutant. In addition, cell elongation with increased cortical actin polymerization which is caused by TNF-*α*-mediated actin filament remodeling is prominent in cells expressing the MK2-K339R SUMOylation defective mutant, compared to WT-MK2, confirming a negative effect of MK2 SUMOylation on TNF-*α*-mediated actin filament remodeling and subsequent EC elongation. Therefore, under TNF-*α*, the decreased actin filament dynamics by sustained inhibition of MK2 kinase activity in the dominant negative (DN)-MK2 and/or the increased actin polymerization by sustained activation of MK2 kinase in the WT-MK2 can inhibit cell movement by deregulating the coordinated “on-off” role of MK2 on actin dynamics [73]. 

MK2 kinase activity regulates not only cell migration but also cytokine production. Studies using MK2-kinase deficient cells demonstrate a central role of MK2 in the production of inflammatory cytokines such as TNF-*α*, IL-1*β*, MIP-1*α*, IL-8, IL-6, and INF-*γ* [74–77]. The involvement of MK2 in upregulating NF-*κ*B target genes VCAM-1 and MCP-1 has also been documented [78]. Therefore, the inhibitory effect of MK2 SUMOylation on its kinase activity may have anti-inflammatory effect in ECs. 

## 7. Potential Effects of Medications Targeting**** Endothelial Inflammation on Protein**** SUMOylation 

### 7.1. Statins

Statins (HMG-CoA reductase inhibitors) are known to reduce low density lipoprotein cholesterol levels by inhibiting the 3-hydroxy-3-methelglutaryl coenzyme A reductase. Numerous studies on statins have been performed, and numerous pleiotropic effects of statins, beyond their cholesterol reduction properties, have been described [79–82]. The inhibition of NADPH oxidase activity was demonstrated as a major mechanism for statins' pleiotropic effects [79]. In particular, statins can inhibit endothelial inflammation [81, 83, 84]. It has been reported that atorvastatin inhibits inflammation in vascular smooth muscle cells and mononuclear cells through the inhibition of NF-*κ*B activity and chemokine gene expression [85]. In addition, the inhibition of NF-*κ*B activity mediated by atorvastatin can improve PPAR signaling in cardiac hypertrophy [86]. While SUMOylation of PPAR*γ* at Lys-107 inhibits its transcriptional activity and increases NF*κ*B activity, statins can improve PPAR signaling and reduce NF-*κ*B activity. These data open a potential, yet to be elucidated, of the linkage between the statins' pleiotropic effects and endothelial inflammation, possibly, through inhibiting PPAR SUMOylation. 

### 7.2. ACE Inhibitors

The beneficial clinical effects of angiotensin-converting enzyme inhibitors (ACEI) have been indicated in many studies [87]. ACEI improve EC function through several mechanisms such as lowering Angiotensin II (Ang II), increasing bradykinin [88], and altering mechanisms that regulate NADPH oxidase activity [89]. Advanced Glycation Products (AGE) have been suggested to play a role in NADPH oxidase signaling, which results in the increased levels of ROS, matrix metalloproteinase (MT-MMP1 and MMP9), monocyte chemoattractant protein-1 (MCP-1), as well as plasminogen activator inhibitor-1 (PAI-1) [90, 91]. However, temocaprilat (ACEI) inhibited all of these AGE-mediated effects [91]. Previously, we reported that AGE and H_2_O_2_ induce endogenous ERK5 SUMOylation [45], suggesting a possible inhibitory effect of ACEI on AGE-induced endogenous ERK5 SUMOylation in reducing endothelial inflammation (Figure 4).

### 7.3. Antioxidant Vitamins

Although the antioxidant properties of vitamins have been reported both *in vitro *and *in vivo* [92, 93], the beneficial clinical effects of vitamins are contradictory. Stephens et al. demonstrated a significant decrease in cardiovascular incidence in patients received vitamin E in the CHAOS (Cambridge Heart Antioxidant Study) clinical trial [94]. However, Harrison et al. indicated that scavenging ROS by exogenous antioxidants is not effective in preventing cardiovascular disease development [95]. In addition, many clinical studies (HOPE, GISSI, and HPS) have not confirmed the protective effects of vitamin E on major cardiovascular events, which was nicely reviewed and summarized by Schramm [96]. The failed promise of antioxidant vitamins suggests that our current concept of oxidative stress need to be revised, and many aspects need to be taken into account [96] for the implications of antioxidant vitamins. 

## 8. ROS, Inflammation, and Cardiac Events in**** Clinical Studies

### 8.1. NADPH Oxidase and Polymorphism

An increasing body of evidence has implicated the role of oxidative stress in atherosclerotic development through regulation of multiple signaling pathways that associates with vascular inflammation [97, 98]. Oxidative stress is the term used to describe the imbalance between producing and removing ROS within a biological system. A major source of ROS within the vasculature is the reduced nicotinamide adenine dinucleotide/nicotinamide adenine dinucleotide phosphate (NAD(P)H) oxidase system. NAD(P)H oxidase is a membrane-associated enzyme, consisting of five subunits that catalyze transfer of an electron to molecular oxygen using NADH or NADPH as the electron donor. Among the five subunits, the 22-kDa NAD(P)H oxidase p22-phox subunit has a polymorphic site on exon 4 which is considered to be the most interesting due to its ability to change NAD(P)H enzyme structure and activity. This polymorphism, C_242_T, is a point of mutation that causes the replacement of histidine by tyrosine at amino acid 72 of the protein, which affects one of the heme binding sites essential for the NAD(P)H enzyme activity [99, 100]. Recent study has found that patients with the NAD(P)H oxidase p22-phox subunit containing T allele on the C_242_T single-nucleotide polymorphism (SNP) instead of C allele are at lower risk for recurrent coronary events than the patients with the C allele [101, 102]. The T allele has been shown to associate with the reduced NAD(P)H enzyme activity, which results in a decrease in vascular peroxidase production [102]. In addition, the T allele also increases the oxidation of high-density lipoprotein (HDL) by altering the redox state in the vasculature [103]. 

### 8.2. Association of the C_242_T SNP of the NAD(P)H Oxidase p22-phox Subunit with CVD Risk in Postinfarction Patients with Concurrently High Levels of HDL Cholesterol and CRP

High levels of HDL cholesterol (HDL-C) are well known to be inversely related to cardiovascular disease (CVD) risk; however, evidence is accumulating indicating that HDL functionality is also important in the protective effects of HDL particles [104–106]. In addition to important roles in reverse cholesterol transport (RCT), HDL particles have additional protective roles including preservation of endothelial function, protection against thrombotic events, and resistance against the inflammatory and oxidative stress-related injury and alterations to the vascular wall and lipoprotein particles. However at the same time, there is growing recognition that atheroprotective effects of HDL can degrade and actually undergo dysfunctional transformation resulting in proatherogenic HDL especially in the setting of inflammation and oxidative stress [107–111].

To explore manifestations of potential HDL dysfunction in the establishment of CVD risk, we have investigated relationships of HDL-C with CVD risk in human populations. We have performed epidemiologic studies specifically focused on HDL-C in the setting of systemic inflammation. To do this, we have studied individuals with concurrently high levels of HDL-C and CRP. Individuals with high levels of HDL-C were chosen to minimize potential confounding effects related to well-known risk associations with low levels of HDL-C, while high CRP levels were chosen as an indicator of systemic inflammation. In terms of high HDL-C, it is notable that multiple earlier studies have demonstrated such associations with CVD risk [112–123]. Our approach has been to use functional genetic polymorphisms and biomarker levels as probes to assess risk associations connected with aspects of HDL functionality. Thus, we have shown for subgroups with concurrently high levels of HDL-C and CRP risk associations with various aspects of RCT; that is, in postinfarction patients recurrent coronary risk with the TaqIB polymorphism of *CETP* [124], and in healthy subjects incident coronary risk with the D9N polymorphism of *LPL*, the TaqIB polymorphism of *CETP*, and high levels of apolipoprotein E [125, 126]. As noted above, it is becoming increasingly clear that HDL possesses protective functions beyond those connected with RCT. In this vein, we have shown for the same subgroup of postinfarction patients, risk associations connected with thrombogenesis using the A387P polymorphism of *THBS4* (thrombospondin-4) [127]; for oxidative stress, a major cause of endothelial dysfunction, the C_242_T polymorphism of *CYBA* (p22phox) [101, 127].

In order to perform such studies, we have developed a graphical discovery tool for distinguishing specific high-risk zones of overlap between high HDL-C and high CRP levels that we call outcome event mapping [128]. Outcome event mapping is an exploratory data analysis approach that generates three-dimensional plots of estimated risk (*z*-axis) as a function of two biomarker levels (*x*- and *y*-axes). Novel aspects of the approach include rank transformation of biomarker levels to more evenly distribute points over the bivariate biomarker risk domain, and the coding of outcome events (0—event absent; 1—event present) with application of a surface-smoothing algorithm to generate a smooth surface over the bivariate risk domain such that the height of the surface at any point in the bivariate risk domain is a measure of the estimated outcome rate at that point. The approach has also been extended to accommodate analyses involving binary variables including single-nucleotide polymorphisms in dichotomized form [126]. We have now used the approach in multiple studies [101, 124–131].

**Figure 5:**
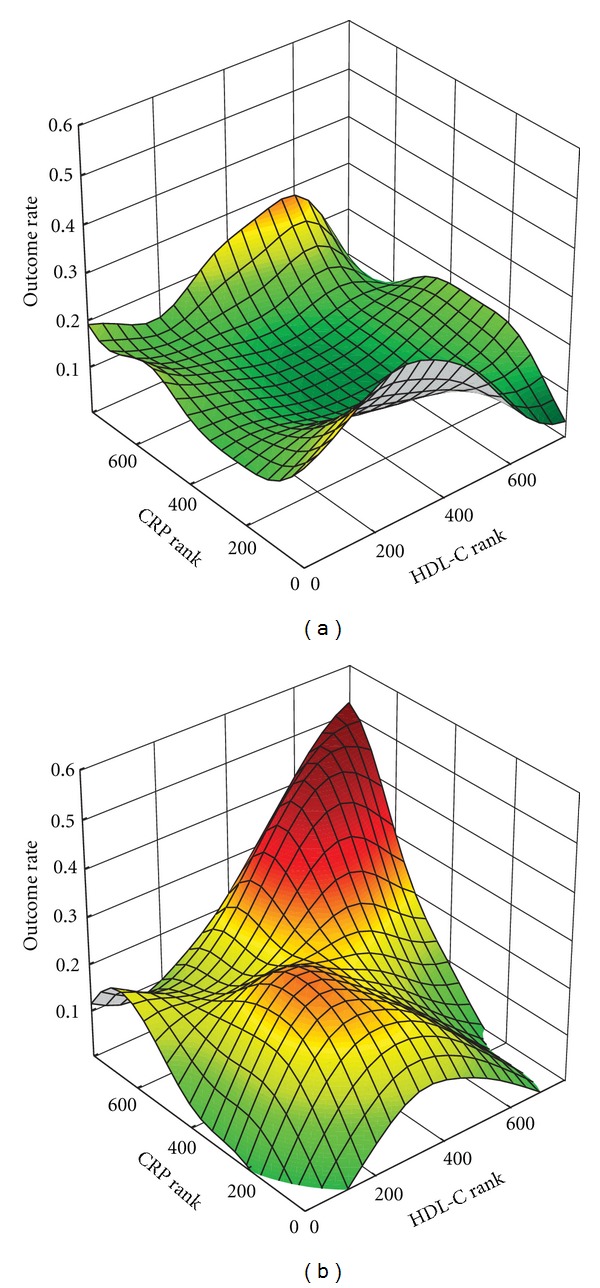
Outcome event mappings in 767 non-diabetic post-infarction patients from the THROMBO study demonstrating estimated outcome event rate as a function of HDL cholesterol and CRP levels (rank-transformed) for (a) carriers of the lower activity T allele (CT plus TT) and (b) homozygotes for the higher activity C allele.

In one such paper, outcome event mapping led to identification at high levels of HDL-C and CRP of a subgroup of postinfarction patients at high risk for recurrent events [127]. Associations of risk with functional genetic polymorphisms connected with HDL activity were then assessed within the subgroup including functionality related to RCT, thrombogenesis, and oxidative stress. Results of multivariable modeling adjusted for significant clinical and laboratory covariates within the subgroup demonstrated significant risk associations for each area; however, results for the C_242_T polymorphism representative of oxidative stress (*CYBA*, p22phox) demonstrated the strongest association (hazard ratio 2.36, 95% CI 1.30–4.17, *P* = 0.004). Specifically, results for the C_242_T polymorphism indicated risk association for patients homozygous for the C allele (normal enzyme activity) in comparison to carriers of the T allele (decreased enzyme activity). Figure 1 presents outcome event maps as a function of HDL-C and CRP: in panel A, for T-allele carriers; in panel B, for C homozygotes. High risk for C-homozygotes is clearly demonstrated at concurrently high levels of HDL-C and CRP by the prominent risk peak at this location (Figure 5(b)) and lack thereof for T-allele carriers (Figure 5(a)).

The observed strong association of risk with the p22phox polymorphism is consistent with the major role of oxidative stress in the development of atherosclerosis extending from the earliest stages of endothelial injury to full-fledged endothelial dysfunction and beyond. This derives from the key role of the generation of ROS in the vasculature, especially superoxide (O_2_^·−^), by NADPH oxidases [132–135]. This process is facilitated by p22phox as an essential activating subunit of NADPH oxidases in the generation of ROS. In terms of endothelial dysfunction, one pathway of ROS generation involves reactive nitrogen species. This starts with depletion of nitric oxide as NADPH oxidase-generated superoxide reacts with it to form peroxynitrite (ONOO^−^). This can subsequently can go on to form additional ROS (hydroxyl radical, HO^·^, and nitrogen dioxide radical, NO_2_^·^). All of these species are known to be potent nitrating agents capable of oxidative modification of biomolecules, including apolipoproteins that may affect function [132, 136–138].

The superoxide generated by the NADPH oxidase system can be the source of additional oxidants in the vasculature through formation of hydrogen peroxide (H_2_O_2_) from superoxide as mediated by superoxide dismutase [138]. In a myeloperoxidase pathway, the enzyme, myeloperoxidase (MPO) found predominantly in neutrophils, monocytes, and macrophages and present in atheroma, mediates the reaction of nitric oxide with hydrogen peroxide to also form the nitrogen dioxide radical [138, 139]. Additionally, MPO mediates the reaction of hydrogen peroxide with chloride ion to form hypochlorous acid (HOCl), another oxidant species potentially affecting molecular function through chlorination. The relevance of these processes to endothelial dysfunction is underscored by the finding that MPO avidly binds to endothelial cells and is subsequently transcytosed to the subendothelial space where its actions, especially with regard to nitric oxide depletion, may occur [139].

With specific regard to HDL, recent work has demonstrated that attack by the aforementioned ROS, generated in large part by actions of the NADPH oxidase system and MPO as noted above, result in nitration and chlorination of specific tyrosine residues and other residues as well on apolipoprotein A-I (apoA-I), the major apolipoprotein constituent of HDL particles and major mediator of multiple HDL functionalities [108, 140, 141]. It is now widely held that such apoA-I modifications can compromise aspects of, for example, RCT including loss of ABCA1-mediated HDL cholesterol acceptor activity and lecithin-cholesterol acyltransferase (LCAT) activation. Additionally, recent evidence suggests that important functions of HDL beyond RCT may be compromised by apoA-I oxidation including antiapoptotic and anti-inflammatory activities [142]. Another aspect of HDL dysfunctional transformation related to oxidative stress may involve resulting modifications in the HDL particle proteome [108]. It is clearly the case that further work must be undertaken to elucidate the actual importance of these and additional processes responsible for dysfunctional transformation of HDL from antiatherogenic to proatherogenic forms.

## 9. Conclusion 

Crucial roles for inflammation in the development of atherosclerosis are evident by an increasing number of studies. Protein SUMOylation has been suggested to regulate a number of biological processes, including inflammation. Therefore, SUMOylation is one of the potential strategies to inhibit inflammation. Because SUMOylation is also required for normal cellular function, targeting the global SUMOylation system might not be an effective strategy to control inflammation. The effect of SUMOylation on inflammation undoubtedly depends on individual proteins that are modified. Therefore, targeting specific SUMOylated proteins that are involved in inflammatory events might be a rational and effective way. We have identified several SUMOylation pathways such as ERK5 SUMOylation, p53 SUMOylation, and MK2 SUMOylation that influence EC inflammation and EC apoptosis, and these pathways have potential relevance to early events of atherosclerosis. These identified SUMOylation pathways could serve as potential targets in reducing EC inflammation. 

In addition to chronic inflammatory disorders, atherosclerosis is recognized as a diffuse, multisystemic disease involving the vasculature, metabolic disorder, and immune systems with various local and systemic manifestations. Thus, merely based on the recognition of a single unstable atherosclerotic plaque to predict the vulnerability of patients to atherosclerosis and CVD is insufficient. Rather, parameters that include a total burden of the atherosclerotic and vulnerable plaques in the aorta, coronary, carotid, and femoral artery as well as blood vulnerability factors is considered important. Currently, attentions are focused on the interaction between inflammation and traditional lipoprotein risk factors. In our study using a subgroup of patients at high risk for recurrent coronary events, we identified high HDL-C as a significant and independent predictor of risk, which also can be employed to evaluate the vulnerability of patients to atherosclerosis and CVD.
